# Research on Underwater Wet Laser Self-Fusion Welding Process and Analysis of Microstructure and Properties of TC4 Titanium Alloy Weld

**DOI:** 10.3390/ma15093380

**Published:** 2022-05-08

**Authors:** Zhihai Cai, Xian Du, Jialei Zhu, Kai Wang, Xiaoxin Zhao, Jun Liu, Jing Li, Jian Liu, Jia Wang, Haidou Wang

**Affiliations:** 1 National Engineering Research Center for Remanufacturing, Army Academy of Armored Forces, Beijing 100072, China; caizhihai2052@163.com (Z.C.); lj54162@126.com (J.L.); gzulijing@163.com (J.L.); xbdliu5899@163.com (J.L.); jasonyan9023@163.com (J.W.); wanghaidou@tsinghua.org.cn (H.W.); 2 Beijing Institute of Petrochemical Technology, Beijing 102617, China; 18810660456@163.com (K.W.); 0020200041@bipt.edu.cn (X.Z.)

**Keywords:** titanium alloy, underwater welding, mechanical properties, residual stress

## Abstract

In order to explore the feasibility of underwater wet laser welding of the TC4 titanium alloy, research on the underwater laser self-fusion welding process was carried out. The weld structure and mechanical properties in both the air environment and the underwater environment were compared and analyzed. The results show that increasing the laser power and reducing the welding speed are beneficial to obtain a larger water depth threshold. Off-focus amount has little effect on water depth threshold; when the laser power is 3000 W and the welding speed is 5 mm/s, and the water depth exceeds 7 mm, a continuous weld cannot be formed. Compared with welding in the air, underwater welding has narrower weld width, smaller heat affected zone and finer crystal grains. The weld structure is mainly composed of α′ martensite and secondary acicular α′ phase, it is distributed in a net basket shape and the grain size at the top of the weld is finer. The hardness of the weld center is above 600 HV0.1, and the residual stress of the underwater welding weld is approximately symmetrically distributed. There is a large tensile stress along the welding direction at the weld, reaching 458 MPa. The larger residual tensile stress leads to the decrease of weld tensile strength, the tensile strength and elongation of the middle sample are only 52% and 77% of the base metal. Furthermore, the fracture mode is typical brittle fracture.

## 1. Introduction

Titanium alloys have a series of outstanding advantages such as high specific strength, good corrosion resistance, stable mechanical properties at high and low temperatures, and potential for welding and machining [1,2,3,4,5,6]. They represent a new structural material with excellent performance after steel materials and aluminum alloys, which are known as the “third metal”, “space metal” and “marine metal”. They have the characteristics of resistance to seawater and salt spray corrosion, and are an ideal marine engineering material. It is widely used in ships, deep-sea exploration, oil and gas development, aerospace, petrochemical, seawater desalination and other fields [7,8,9,10]. In the 1960s, the United States, Russia, Japan, China and others began to study titanium alloys in the field of ships. In 1968, the first all-titanium nuclear power submarine was launched in Russia, which laid its leading position in the construction technology of titanium alloy nuclear submarine [11]. At the same time, Russia was the first country to build titanium alloy pressure hull [12]. With the price of titanium alloy falling, in recent years, China has paid more and more attention to the application of titanium alloy in the marine field, such as the “Jiaolong” deep submersible using titanium alloy shell. At present, there have been many studies on titanium alloy welding on land [13,14,15,16,17], while the complexity and particularity of underwater environment determine that there are relatively few studies on underwater titanium alloy welding technology. Guo Ning et al. [18] prepared an underwater cladding layer of the Ti-6Al-4V titanium alloy by underwater laser wire-feeding cladding technology. The microstructure, grain characteristics, and properties of welds were systematically analyzed, which provides theoretical guidance for the research of titanium alloy underwater welding. However, at present, the theory of titanium alloy wet underwater laser welding is not mature, and further research is still needed.

Underwater welding technology usually consists of three welding methods: dry welding, local dry welding and wet welding [19,20,21]. Compared with the other two methods, underwater wet welding is the cheapest and easiest welding method, and flux-cored wire arc welding is the most widely used. Zhang et al. [22] compared the effects of heat input and droplet transfer on the geometry and microstructure of welds in underwater wet flux-cored wire welding (FCAW). It was confirmed that the content of acicular ferrite and proeutectoid ferrite in underwater weld was less. The droplet transfer was dominated by large droplet rejection, resulting in asymmetry and uneven morphology of weld. Sun et al. [23] explored the influence of ultrasonic on arc stability of underwater wet flux-cored wire welding, and pointed out that the stability of arc was significantly improved after adding ultrasonic. XU et al. [24] observed the dynamic behavior of underwater wet welding pool by X-ray transmission method, and proposed a method to track the position of a point to characterize the stability of the molten pool. It is pointed out that the violent fluctuation of underwater welding pool is caused by the evolution of bubbles (especially the rupture of large bubbles). At the same time, ultrasonic can effectively reduce the size of bubbles in the molten pool, thereby reducing the fluctuation of the molten pool. Fu et al. [25] used the local dry method to weld TC4 titanium alloy underwater. Through the analysis of welding quality, the heat input and defocus distance were optimized. Guo et al. [26] studied the effects of process parameters such as laser power, shielding gas flow and focus position on the welding quality of TC4 titanium alloy by underwater laser welding. Through the optimized process parameters, the formed good welding quality was obtained, and the microstructure was martensite structure. Luo et al. [27] studied the oxidation hardening behavior of the weld in detail by local dry underwater laser welding of cp Ti. It is found that local dry underwater laser welding combined with air assisted purging is a feasible method to obtain complete and smooth weld. Wang et al. [28] designed a mechanical constraint assisted underwater wet welding (MC-UWW) device, and observed the arc bubbles in the process of underwater wet welding using a high-speed camera. The results showed that the mechanical constraint could constrain the arc bubbles near the molten pool, thus providing better protection for welding.

Compared with other welding technologies, underwater laser welding has the advantages of low heat input, fast welding speed, deep penetration, and small deformation. These features make it can overcome the cracks caused by arc welding gas [29]. Moreover, the existing research focuses on the local dry welding process, with complex equipment and poor flexibility. It is necessary to explore the feasibility of wet laser welding. Based on this, this paper takes titanium alloy as the research object, explores the feasibility of underwater wet laser self-fluxing welding, studies the forming behavior and weld performance of underwater welding, and compares it with the weld in air. The effects of underwater environment on the microstructure and mechanical properties of laser welded TC4 were analyzed by XRD, SEM, and EBSD so as to provide scientific basis for underwater welding of titanium alloy.

## 2. Materials and Methods

A TC4 titanium alloy was used as the base metal in the experiment, and its size was 100 mm × 80 mm × 10 mm. The chemical composition provided by the manufacturer is shown in Table 1. Before welding, V_HF_: V_HNO3_: V_H2O_ = 5: 30: 65 mixed solution was used to pickling the welding surface to remove the oxide layer on the surface. When welding the sample directly placed in tap water, the laser beam through the water directly acts on the surface of the substrate for welding.

The IPG YLS-6000 fiber laser was used in the experiment. The maximum output power was 6 kW and the wavelength was 1070 nm. The laser was transmitted through the fiber with a core diameter of 200 μm to the welding joint installed on the six-axis robot. After collimating and focusing, a spot with a diameter of 0.3 mm was obtained for welding. The self-made underwater environment simulation equipment was placed on the mobile welding worktable below the laser welding joint.

**Figure 1:**
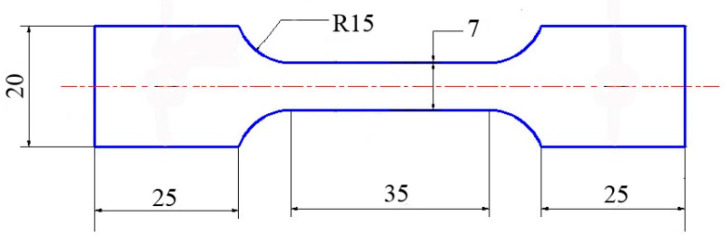
Dimensions (mm) of specimens for tensile tests.

After welding, the metallographic samples were cut out along the direction perpendicular to the weld. After grinding and polishing, the samples were eroded by V_HF_: V_HNO3_: V_H2O_ = 3: 5: 92 mixed solutions, and the microstructure was observed by Olympus GX-51 optical metallographic microscope. The microstructure and micro-area composition of the samples were observed and analyzed by scanning electron microscope (SEM) equipped with energy dispersive spectrometer (EDS). The accelerating voltage (AccV) is 15 kV. The phase structure of weld was analyzed by D8 X-ray diffractometer (XRD) produced by German Bruker company. XRD adopts continuous scanning mode. The incident wavelength was 0.154 nm, the diffraction range was 20°–100°, and the diffraction speed was 2°/min. Oxford Symmetry electron backscatter diffraction (EBSD) was used to characterize the crystal orientation, grain boundary information and grain size in the weld. The accelerating voltage is 20 kV and the scanning step is 0.3 μm. Microhardness was measured by Archimedes Microhardness Tester: tensile test was carried out on the sample by universal testing machine with a loading rate of 1 mm/min to analyze the microscopic characteristics of the fracture. Tensile specimens were machined perpendicular to the welding seam. The dimensions of tensile specimens were designed following AWS D3.6M-2017 Underwater Welding Code (as shown in Figure 1). A hardness test was carried out in accordance with ASTM E 92.

## 3. Results and Analysis

### 3.1. Feasibility Study of Underwater Wet Laser Welding

**Figure 2:**
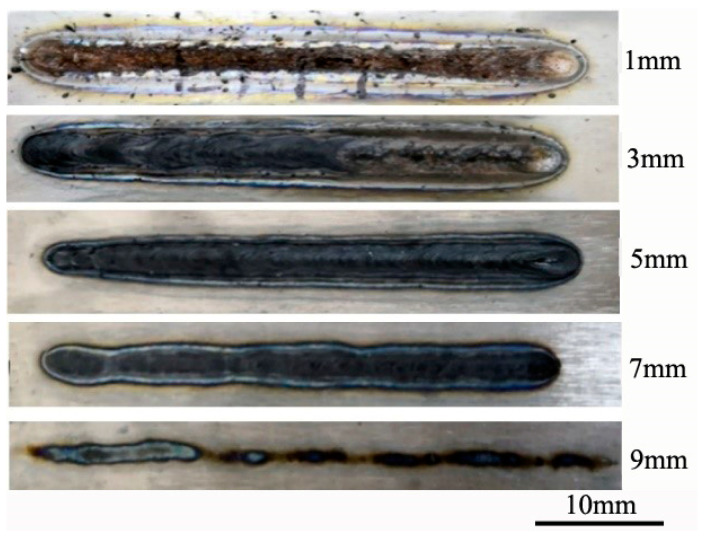
Macroscopic morphology of wet laser welding seam under different water depths.

During underwater laser welding, since the thermal conductivity of water is much higher than that of air, the cooling rate of molten pool is very fast during welding. This phenomenon affects the hydrogen escape in molten pool and is not conducive to the formation of weld. At the same time, water is decomposed under the action of high temperature, increasing the hydrogen content in the molten pool. It is prone to hydrogen embrittlement, white spots, hydrogen pores and cold cracks. With the increase of underwater pressure, the natural convection of molten pool will be enhanced and the ratio of weld depth to width will be reduced, thus affecting the welding quality. In addition, the interaction mechanism between water and laser is complex, resulting in poor forming stability and large power loss [30,31,32]. In order to verify the feasibility of underwater laser wet welding process, 3000 W laser power, 5 mm/s welding speed, and 0 mm off-focus amount were selected to carry out underwater wet laser welding experiments under different water depths. The macroscopic morphology of the weld is shown in Figure 2. It can be seen from the figure that when the water depth is shallow, the weld oxidation is serious. When the water depth threshold exceeds 7 mm, the laser energy is insufficient for stable laser welding. When the water depth is only 1 mm, the water on the surface of the substrate is rapidly vaporized under the action of the laser. At this time, the underwater laser welding is carried out in an unprotected environment. The molten pool is directly exposed to the surrounding environment, and the oxygen absorption of liquid metal titanium at high temperature is seriously oxidized. When the water depth increases to 3 mm, the pressure of water in the welding area increases. The vaporization pressure caused by laser is not enough to completely drain the water in the welding area. The welding area is surrounded by bubbles formed by evaporation, this prevents oxygen in the surrounding environment from entering the welding area, so the front of the weld is not oxidized. But as the welding proceeds, the heat accumulates in the matrix. The evaporation of water becomes more and more intense, until the evaporation bubbles completely drain the water from the welding area. The molten pool is exposed to the surrounding air, causing oxidation at the back of the weld. The water depth continues to increase to 5 mm, the bubbles are stable in the welding process, the weld is uniform and beautiful, the surface is black, and it shows a bright metallic luster after polishing. When the water depth continues to increase to 7 mm, due to the strong metal gasification, the liquid metal in the molten pool migrates counter-laser axially along the keyhole wall, causing humps on the surface of the molten pool. At the same time, due to the rapid solidification of the laser welding molten pool, the molten metal cannot be backfilled immediately, resulting in a bite edge defect in the weld. When the water depth increases to 9 mm, the plasma formed by water ionization forms a strong shielding effect on the laser, resulting in welding failure.

### 3.2. Effect of Process Parameters on Welding Forming

**Figure 3:**
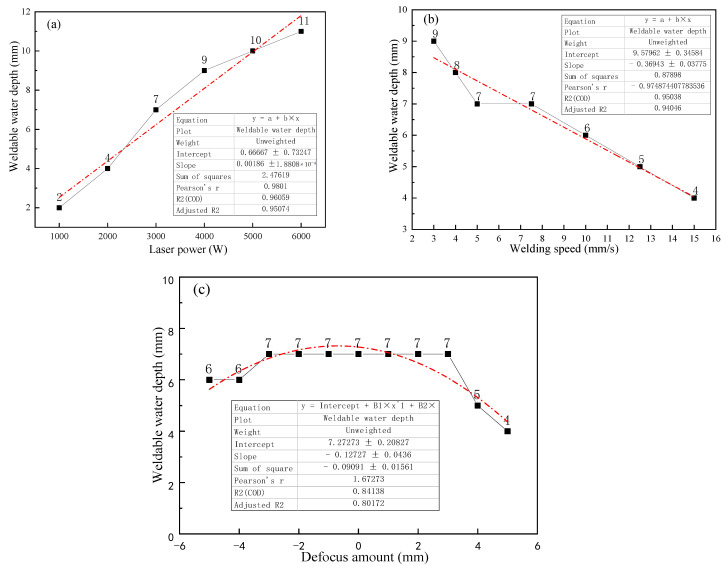
Effect of process parameters on welding depth: (**a**) laser power; (**b**) welding speed; (**c**) off-focus amount.

There are many factors affecting the water depth threshold of underwater laser welding. The effects of laser power, welding speed, off-focus amount and other process parameters are studied. The results are shown in Figure 3. It can be seen from the Figure 3a that the laser power has a great influence on the welding depth threshold. With the increase of laser power, the water depth threshold increases correspondingly. There is a positive correlation between the laser power and welding depth. When the laser power increases from 3000 W to 6000 W, the water depth threshold increases only 4 mm. As described in Figure 3b, the welding speed determines the time of laser interaction with the substrate in the welding process, and then determines the total energy of the substrate obtained from the laser in the welding process. With the increase of welding speed, the interaction time between laser and metal becomes shorter, and the energy obtained by the base metal decreases. When the welding speed is too fast, the molten pool temperature will be insufficient. However, if the welding speed is too slow, it will cause excessive heat input, increase the heat-affected zone, and coarsen the crystal grains.

As shown in Figure 3c, the off-focus amount has little effect on the water depth threshold. When the off-focus amount changes between +3 and −3 mm, the water depth threshold does not change. However, if the off-focus amount is too large, the power density will decrease. So, the water depth threshold of welding will also decrease. In summary, laser welding process parameters have a certain impact on the welding water depth threshold of TC4 titanium alloy, but the impact is limited.

**Figure 4:**
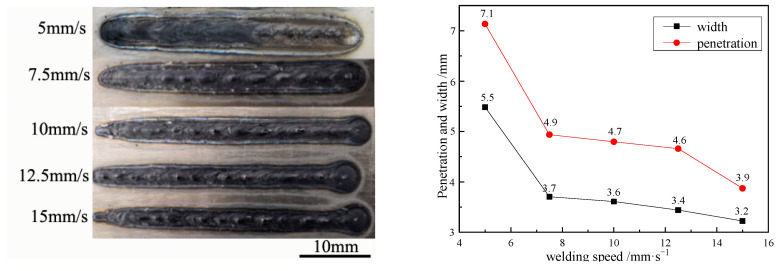
Effect of welding speed on weld macromorphology, penetration depth, and width.

When the fixed laser power is 3000 W and the water depth is 3 mm, the influence of welding speed on the macro morphology, penetration depth and penetration width of the weld is further analyzed. The results are shown in Figure 4. It can be seen from the figure that when the welding speed is 5 mm/s, the oxidation phenomenon occurs at the rear of the weld. With the increase of the welding speed, the total laser input heat of the weld decreases for unit length. The temperature of the molten pool decreases, and the evaporation and vaporization of the surrounding water decreases. The formed bubbles are not completely discharged from the water in the welding area. Therefore, there is no oxidation in the weld with high speed. With the increase of welding speed, the cooling and solidification of molten pool is more rapid, this is not conducive to the flow of molten pool. It is easy to form hump-shaped defects in the weld, resulting in an uneven weld. At the same time, from the influence of welding speed on the penetration and width, it is more obvious that with the increase of welding speed, the penetration and width decrease. When the welding speed is 5 mm/s, the penetration is about 7.13 mm, and when the welding speed increases to 15 mm/s, the penetration is 3.87 mm (about 54% of the former).

**Figure 5:**
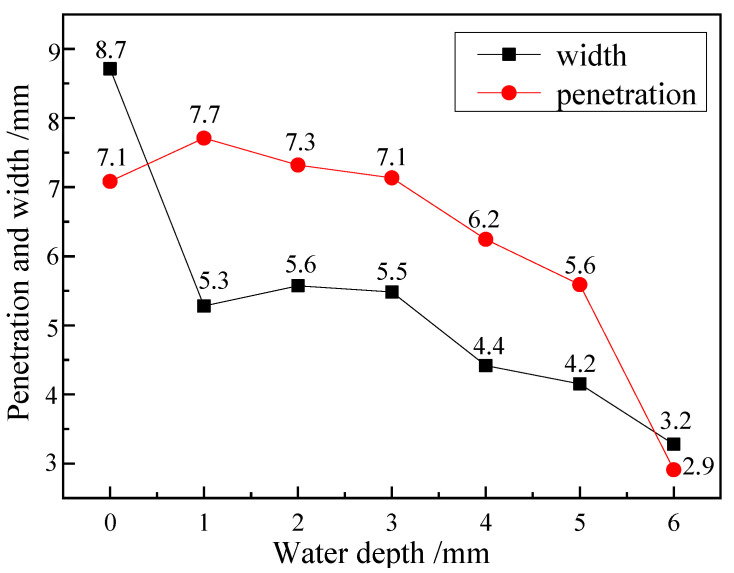
The weld penetration and weld width under different water depths.

When the laser power is 3000 W and the welding speed is 5 mm/s, the weld depth and width at different water depths are shown in Figure 5. It can be seen from the figure that there is little difference in the penetration depth between the shallow water and the air welding, indicating that the laser energy loss is low in shallow underwater welding. However, with the increase of water depth, the penetration depth gradually decreases. When the water depth exceeds 5 mm, the penetration depth decreases sharply, indicating that a considerable part of the energy is absorbed, scattered or refracted when the laser passes through the plasma generated in the welding process. When the water depth is more than 7 mm, the laser can hardly penetrate the plasma. The laser energy reaching the metal surface is not enough to make the metal melt into small holes, and the laser deep penetration welding cannot continue.

### 3.3. Analysis of Weld Cross-Section Morphology

**Figure 6:**
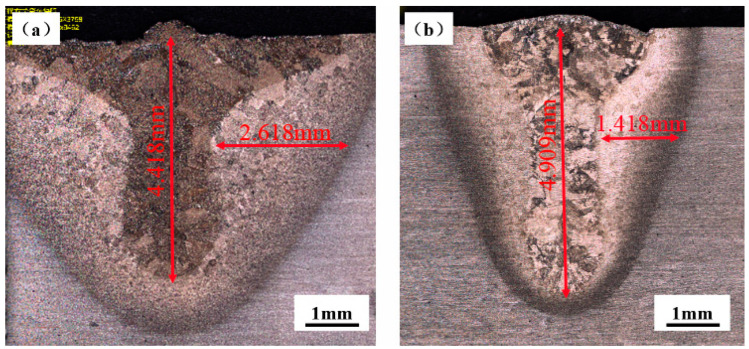
Cross section of air and underwater welds: (**a**) welding in air; (**b**) underwater welding.

The cross-section morphology of underwater welding seam was analyzed and compared with that in air, as shown in Figure 6. The laser power is 3000 W and the welding speed is 5 mm/s, both “air welding” and “underwater welding”. TC4 is α + β titanium alloy at room temperature. During the welding process, the molten pool metal is heated and melted, reaching the transition temperature of α phase to β phase, so all of them are transformed into β phase [33,34]. During the cooling and solidification process, the temperature of molten pool presents a gradient distribution. The temperature decreases gradually from the center of molten pool to the heat affected zone and then to the TC4 matrix. At the junction of the molten pool and the heat affected zone, the liquid metal in the molten pool directly contacts with the grains of the TC4 matrix and is completely wet. During the cooling process, the liquid metal takes the grain of TC4 matrix as the nucleation matrix, and grows along the original crystal orientation. The β columnar crystal is epitaxially grown to the weld center on the semi-melted metal near the fusion line. Although the welds in air and underwater show typical ‘nail head’ shape, the ‘nail head’ and heat affected zone of underwater welding welds are smaller than that of air welding, which is mainly caused by two reasons. One is that the plasma formed during underwater welding is constrained in a relatively small space by the pressure of surrounding water, so the plasma volume is small. The other is that water has a strong cooling effect on the molten pool, resulting in a certain heat loss.

**Figure 7:**
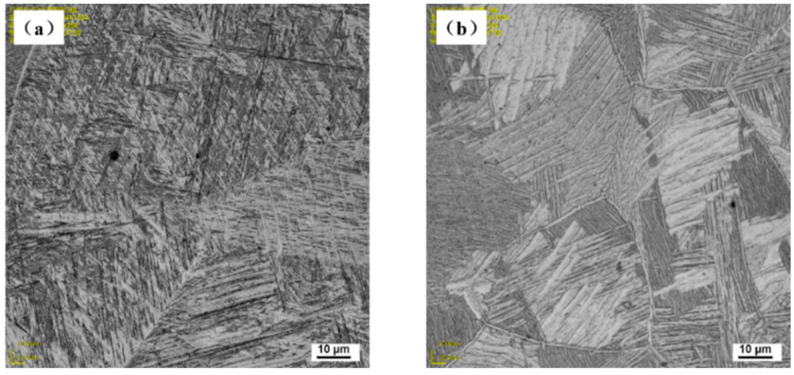
Metallography in the middle of weld: (**a**) welding in air; (**b**) underwater welding.

**Figure 8:**
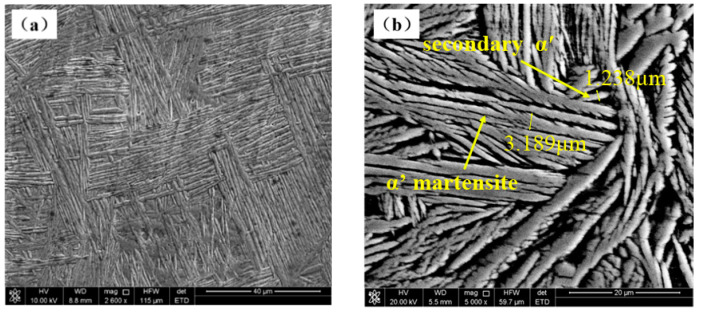
Net basket structure in underwater welds: (**a**) net basket structure; (**b**) local magnification.

When welding in air, the weld is mainly cooled by the thermal radiation to the surrounding air and the heat conduction to the base metal. The cooling rate of the molten pool is relatively slow, and the diffusion type transformation of β phase→α phase occurs in the cooling process. Finally, the coarse original grain β phase is formed in the molten pool, and the α phase is distributed in a network on the original β grain boundary. The α phase in the original β grain is distributed in a sheet, this is a typical Widmanstatten structure, as shown in Figure 7a. In underwater wet laser welding, due to the rapid cooling effect of the surrounding water environment on the matrix and the molten pool, the molten pool rapidly cools and solidifies in a very short time. The β phase forms α′ martensite through non-diffusion transformation, that is, the β phase→α′ martensite transformation occurs. Therefore, the microstructure is different from that of the weld in the air, as shown in Figure 7b. The typical Widmanstatten structure is also formed in the underwater wet welding weld, but the grain size is smaller. This is due to the fact that during welding in the air, the molten pool mainly dissipates heat through the heat conduction with the base metal and the thermal radiation with the surface air. During the wet underwater welding, the molten pool not only conducts heat conduction to the base metal, but also exchanges heat with the surrounding water. Therefore, the cooling rate is faster and the grain size in the weld is smaller. During the rapid cooling and solidification process of underwater wet laser welding, α′ martensite phase nucleates at the internal boundary of the original β grain and grows parallel to the internal of β grain, and finally runs through the whole β grain. The secondary acicular α′ was formed in α′. Between the martensitic phases, the width is only about 1–2 microns. These fine secondary acicular α′ phase showed different orientations with the α′ martensite phase. Finally, the basket-like microstructure was formed, as shown in Figure 8. [25]

**Figure 9:**
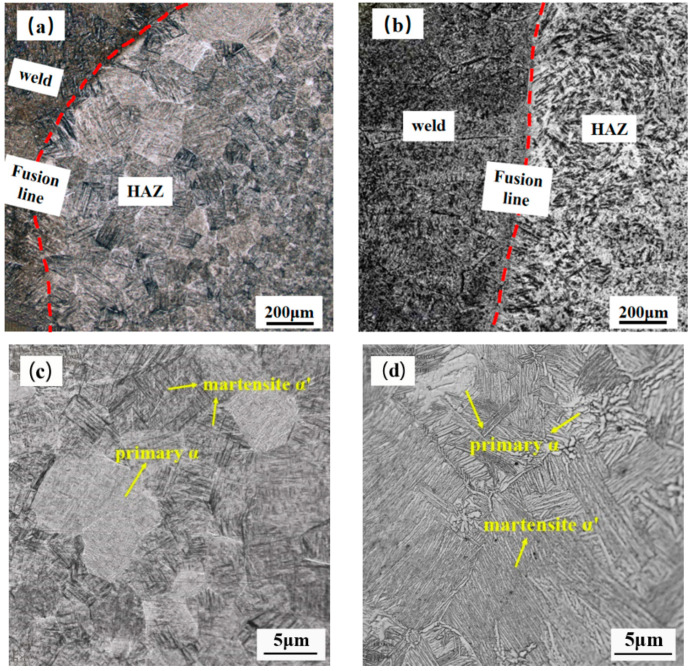
Metallograph near fusion line: (**a**) welding in air; (**b**) underwater welding; (**c**) local magnification, welding in air; (**d**) local magnification, underwater welding.

The metallographic organization near the fusion line is shown in Figure 9. Figure 9c,d are local magnifications of Figure 9a,b, respectively. The heat-affected zone is composed of α′ martensite and the initial α phase. The reason is that the heat affected zone is second only to the weld during the welding process. The heat affected zone temperature increases with the decrease of the distance from the weld. In the heating process of the heat affected zone near the weld, the TC4 substrate is heated. The highest temperature of the TC4 substrate reaches the transformation temperature of α phase→β phase. Then, the β single-phase zone is formed. During the cooling process, β phase forms α′ martensite through shear. During the heating process of the heat-affected zone far away from the weld, the temperature is relatively low. Only part of the α phase undergoes the allotropic transformation from α phase to β phase, forming a dual phase zone of β + α. During the cooling process, β phase forms α′ martensite phase through shear. The α phase is not transformed. So, a mixed structure of α′ martensite phase and initial α phase is formed.

### 3.4. Microstructure Analysis of Weld

**Figure 10:**
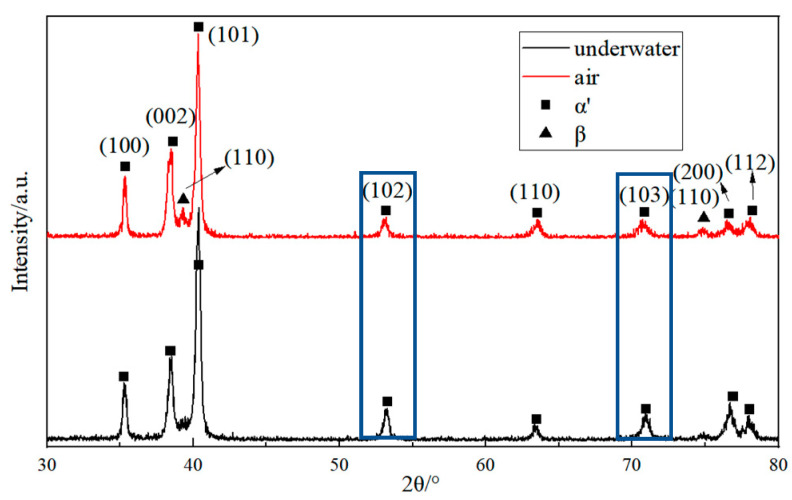
The XRD analysis pattern of TC4 alloy welds in air and underwater.

The XRD analysis pattern of TC4 alloy welds in air and underwater is shown in Figure 10. It can be seen from the figure that the weld of air welding and underwater welding are mainly composed of α′ and β phase. The content of α′ phase underwater welding is obviously higher than that of weld in air. Due to the fast-cooling rate of molten pool in underwater environment, the β phase transformation is reduced at this time, so the weld of underwater welding obtains more of an α′ phase.

**Figure 11:**
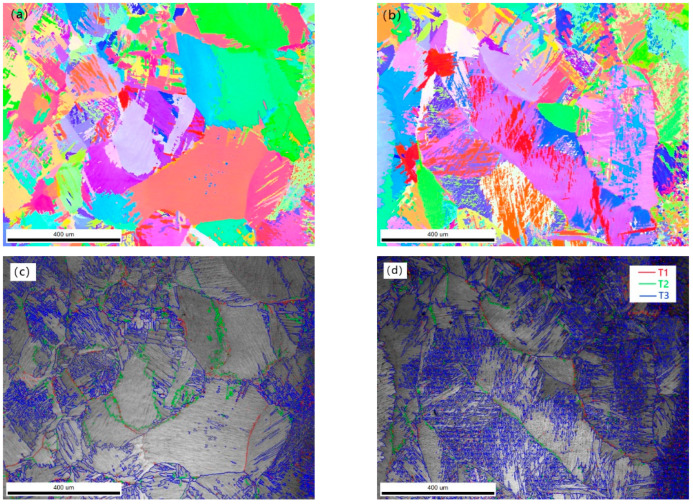

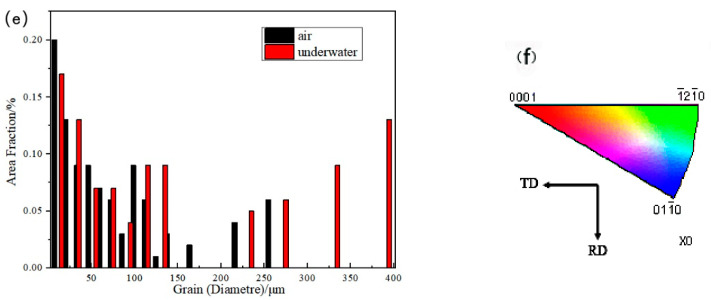
Underwater welding weld EBSD. (**a**) the IPF map of grains at the top of the weld; (**b**) the IPF map of grains at the bottom of the weld; (**c**) grain boundaries at the top of the weld; (**d**) the grain boundaries at the bottom of the weld; (**e**) grain sizes comparison (**f**) IPFs in the ND.

In order to further understand the size and orientation of the grains in the weld, EBSD analysis of the underwater wet welding weld is carried out. The distribution of grains, grain boundaries and grain sizes at the top and bottom of the underwater wet welding weld are shown in Figure 11. The β grains on the top of the molten pool are equiaxed. The grains between the equiaxed grains are clearly visible. The acicular α′ martensite inside the grains has different orientations. Acicular in grain α′ martensite has different orientations, mainly (0001)//Z α′ and (1214)//Z α′ staggered distribution, primary α (1210)//Z distributed in blocks. Bottom of the weld (0001)//Z α′ was increased content, primary (1210)//Z α was decreased. The β columnar crystals on the bottom of the molten pool are massive, as shown in Figure 11b. The grain size of β at the top is fine equiaxed crystal, which is consistent with the grain size measurement results in Figure 11e. The phase transformation β→α′ during cooling, is typically governed by Burgers orientation relationship. The resultant orientation relationship between β and α′ phases provide specific crystallographic α′ variants. The distributions of the various grain boundaries including low-angle grain boundaries (LABs < 10°), two types of variant boundaries and other general high-angle grain boundaries (GHABs) for the samples are shown in Figure 11c,d. It is indicated that major part (more than 2/3) of grain boundaries belong to variant boundaries, in which the variant boundary of 60°/[1120] misorientation has indicated by T1(blue lines in figure). This is commonly observed in the distribution of martensite variant boundaries in titanium alloys. It should be noted that a fraction of boundaries does not follow any misorientation angle/axis pairs associated with the Burgers orientation relationship, namely GHABs, as indicated by red lines. The other one is from the random impingement of two crystallographic variants transformed from two distinct neighboring prior β grains as illustrated by the green lines. Due to the martensitic transformation is to complete the lattice reconstruction in the process of crystal shear, the lattice and the parent β approximately follow the burgers relation: (0001)α′//(001)β, (1210)α′//(111)β.

### 3.5. Analysis of the Mechanical Properties of Welds

**Figure 12:**
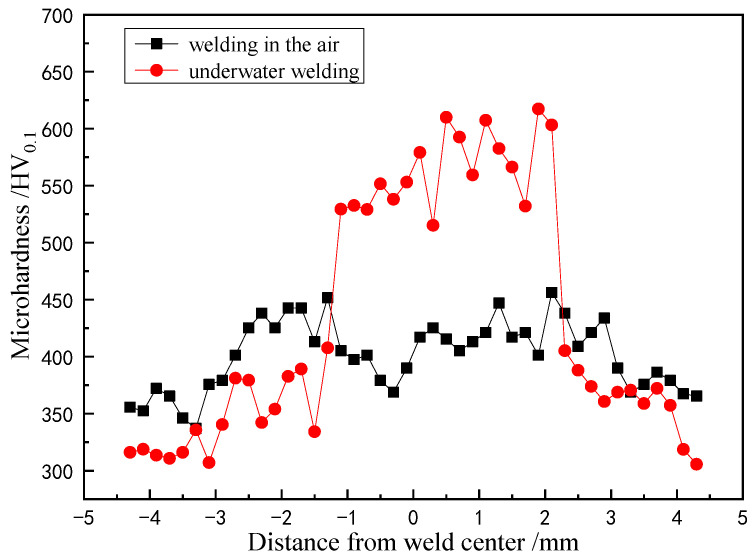
Microhardness distribution curve of air and underwater welding welds.

The microhardness distribution curve of air and underwater welding welds is shown in Figure 12. It can be seen from the figure that the microhardness of underwater weld and heat-affected zones is significantly higher than that of the TC4 substrate. The hardness of the center area of the weld is the highest, reaching more than 600 HV0.1. The hardness of the center area of the weld is much higher than the microhardness of 400 HV0.1 at the center of the air weld. The microhardness decreases with the increase of the distance from the weld center. The microhardness of the underwater heat-affected zone is lower than 400 HV0.1; while the microhardness of the heat-affected zone of air welding is higher than that of the weld center, reaching 450 HV0.1. The above-mentioned difference in hardness is mainly caused by the difference in weld structure. The weld structure of underwater wet laser welding is a Widmanstatten structure. The weld structure of underwater wet laser welding is mainly composed of α′ martensite phase and secondary acicular α′ phase. The heat-affected zone structure is a mixed structure of α′ martensite phase and initial α phase. The substrate microstructure is a dual phase structure of α + β. Although the weld structure in air is also a Widmanstatten structure, β grains are distributed in lamellar α phase. Among these structures, the hardness of α′ martensite phase is the largest. The hardness increases with the increase of the α′ martensite phase content. Therefore, the microhardness of the underwater weld is the largest. The hardness of the heat affected zone is also larger due to the presence of some α′ martensite phase, while the hardness of the weld welded in air is smaller due to the absence of α′ martensite phase.

**Figure 13:**
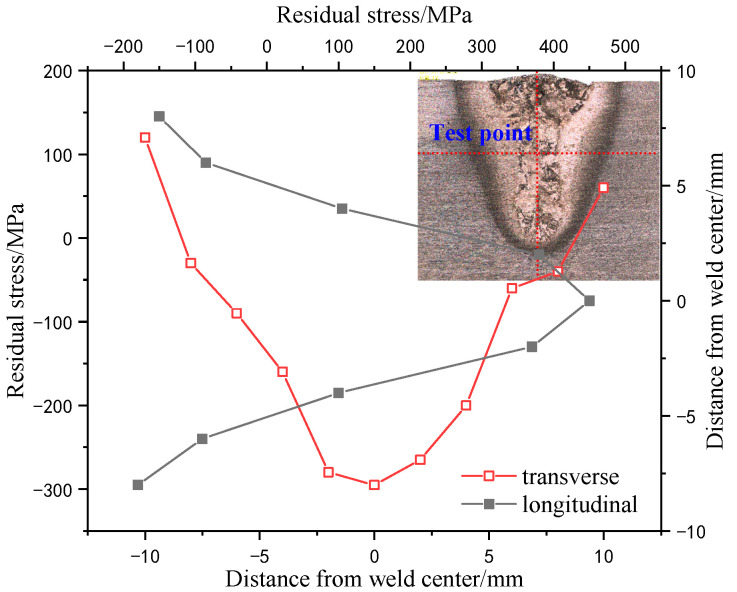
Distribution of transverse and longitudinal residual stress.

**Figure 14:**
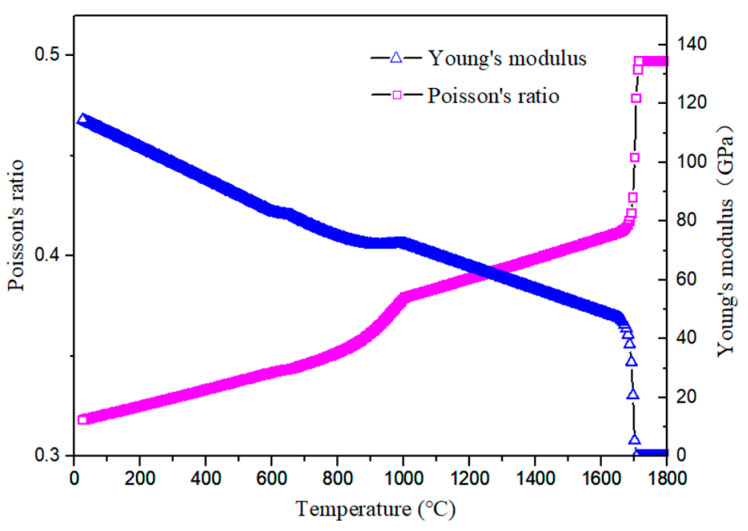
Variation of Young’s modulus and Poisson’s ratio with temperature.

The distributions of residual stresses in tangential and longitudinal directions of underwater welding weld are shown in Figure 13 and Figure 14 respectively. Residual stress is approximately distributed symmetrically. It can be seen from Figure 13 that the residual stress at the center of the weld appears as a compressive stress with a magnitude of −295 M. The residual stress gradually becomes a tensile stress away from the weld. In the longitudinal direction of the weld, the residual stress distribution appears as a larger tensile stress at the center of the weld, reaching 458 MPa. The residual stress appears as a smaller compressive stress far away from the weld. This phenomenon is caused by the uneven temperature distribution on the surface and bottom of the molten pool during underwater welding. Since the young’s modulus and Poisson’s ratio of TC4 titanium alloy vary greatly with different temperature, the thermoplasticity in the weld section is also uneven.

**Figure 15:**
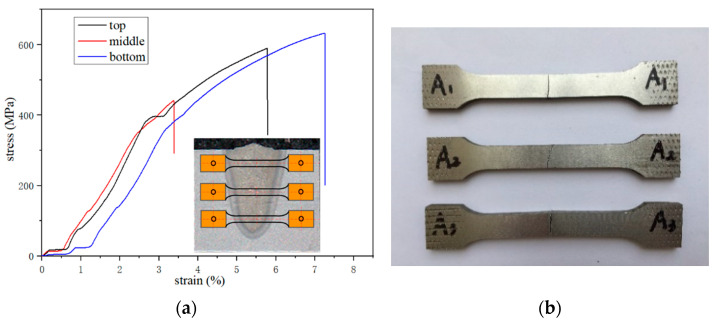
Tensile test: (**a**) stress-strain curves; (**b**) tensile specimens.

**Figure 16:**
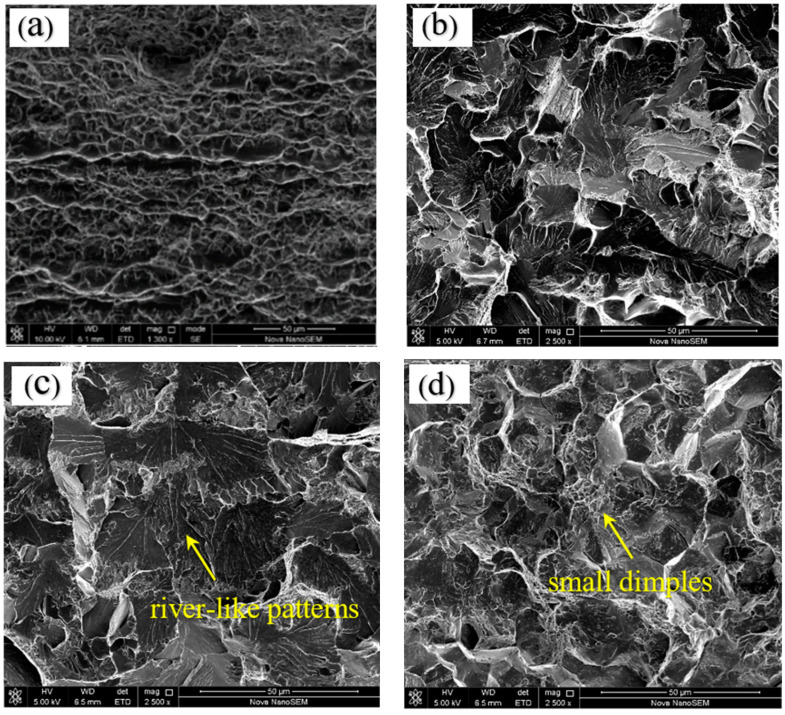
Fracture of tensile specimen: (**a**) TC4 substrate; (**b**) top of the weld, (**c**) middle of the weld, (**d**) bottom of the weld.

The top, middle, and bottom of underwater laser weld were stretched test respectively. In order to reduce accidental factors, measure three times under each welding condition, and take the average value as the final result. All of the tensile specimens fractured at the weld. The tensile results are shown in Table 2 and Figure 15. The tensile strength of the TC4 matrix reaches 932 MPa. The elongation after fracture reaches 14.6%. The matrix is a typical ductile fracture. The tensile strength and elongation of underwater weld decreased, but the tensile specimens at the bottom and top of the weld obtained higher strength and elongation, while the tensile strength and elongation of the middle specimen decreased the most, with the tensile strength and elongation reduced by 52% and 77%, respectively. The reason for this phenomenon is related to the distribution of residual stress. It can be seen from Figure 13 that the residual stress in the middle of the weld is tensile stress, reaching 458 MPa. It reaches the yield limit in advance during the load bearing process, resulting in the early failure of the material. The top and bottom of the weld show compressive stress, which improves the yield limit of the material and helps to improve the strength of the material. In addition, the elongation also shows the same trend as the tensile strength, and the compressive stress improves the plastic deformation ability of the weld. Observe the tensile fracture morphology of different areas of base metal and weld, and fracture morphology are shown in Figure 16. As can be seen from the figure that the TC4 matrix shows a typical dimple fracture mode. The fracture surfaces in Figure 16b,c are relatively flush without obvious necking, and the river pattern in Figure 16c is obvious, which is a typical cleavage fracture feature. A large number of small dimples appear on the grain boundary on the fracture surface in Figure 16d. The fractured grain boundary is coarser than that in Figure 16b,c, showing the fracture characteristics of mixed toughness and brittleness.

## 4. Conclusions

The research on underwater wet laser self-fusion welding of TC4 titanium alloy was carried out. Influence of process parameters on weld formability was discussed. The microstructure and mechanical properties of the welds in an air environment and underwater environment were compared and analyzed. The conclusion is as follows:

(1) Under certain conditions, underwater wet laser welding is feasible. When the laser power is 1000~6000 W and the welding speed is 3–15 mm/s, increasing the power and reducing the welding speed are beneficial to obtain a larger water depth threshold; the off-focus value has little effect on the water depth threshold. The weld forming performance becomes worse as the water depth of the workpiece surface increases. When the water depth exceeds 7 mm, a continuous weld cannot be formed.
(2) Underwater welding weld and air welding weld are typical ‘nail type’ morphology. Both are constrained by the underwater plasma and rapid cooling. The underwater welding seam has a narrow melting width, a small heat-affected zone, and a finer grain. The αphase on the β grain boundary of the air weld is distributed in a network shape. The αphase inside the β grain is distributed in a flaky shape; underwater weld α′ martensite runs through the whole β grain, forming secondary acicular α′ phase between α′ martensite phase, forming basketweave structure. The grain size at the top of weld is finer.
(3) Compared with the welding in the air, the α′ phase content of underwater welding is significantly higher. The center hardness of the underwater welding seam reaches more than 600 HV0.1. The residual stress of the welding seam is approximately symmetrically distributed. There is a large tensile stress at the welding seam along the welding direction (longitudinal), reaching 458 MPa. The residual stress appears as a small compressive stress with the increase of the distance from the weld. The existence of compressive stress improves the yield limit of the weld and helps to improve the strength. The distribution of residual stress also affects the fracture morphology. The existence of compressive stress improves the plastic deformation ability of weld.

## Figures and Tables

**Table 1:** Chemical composition of the TC4 titanium alloy (mass fraction, %).

Element	Al	V	C	O	N	Ti
TC4	6.06	3.92	0.013	0.15	0.014	Margin

**Table 2:** Tensile results of different tensile specimens.

	Tensile Strength/MPa	Elongation/%
TC4 substrate	932	14.6
underwater weld (top)	591	5.8
underwater weld (middle)	439	3.3
underwater weld (bottom)	618	7.2

## Data Availability

Not applicable.
